# Cross-cultural adaptation and validation of the Arabic version of the simple shoulder test in the United Arab Emirates

**DOI:** 10.1371/journal.pone.0267885

**Published:** 2022-05-04

**Authors:** Tamer Shousha, Fatima Alowais, Ashokan Arumugam

**Affiliations:** 1 Department of Physiotherapy, College of Health sciences, University of Sharjah, Sharjah, UAE; 2 Neuromusculoskeletal Rehabilitation Research Group, Research Institute of Medical and Health Sciences, University of Sharjah, Sharjah, UAE; 3 Department of Physical Therapy for Musculoskeletal Disordered and its Surgery, Faculty of Physical Therapy, Cairo University, Cairo, Egypt; 4 Department of Physiotherapy, Qassimi Hospital, Sharjah, UAE; 5 Sustainable Engineering Asset Management Research Group, RISE-Research Institute of Sciences and Engineering, University of Sharjah, Sharjah, UAE; University of Auckland, NEW ZEALAND

## Abstract

**Background:**

The Simple Shoulder Test (SST) is a simple and short patient-reported outcome measuring functional limitations of the affected shoulder in patients with shoulder dysfunction. Although it is widely used in different clinical cultures, literature review to date revealed that the SST has not been yet translated nor validated in the Arabic language.

**Research objectives:**

To translate, culturally adapt, and validate the Arabic version of the Simple Shoulder Test (SST).

**Methodology:**

A forward-backward translation method was adopted. One hundred and forty-one patients with shoulder pain were recruited for psychometric analysis based on the inclusion criteria. The test–retest reliability of the Arabic SST (ASST), pain, disability and total scores were assessed using intraclass correlation coefficients (ICC). The construct validity of the ASST was tested by Spearman rank coefficients through comparing the Arabic SST scores to the severity of shoulder pain measured using the visual analogue scale (VAS) and the Arabic version of the Shoulder Pain and Disability Index (SPADI). Internal consistency was assessed by the Cronbach’s alpha.

**Findings:**

One hundred and forty participants (60 males and 80 females) with a mean (Standard Deviation) age of 39.3 (4.9) years participated in the study. The ICCs for score of ASST were reported high; pain 0.84 (0.78–0.93), disability 0.96 (0.93–0.97) and total score 0.95 (0.91–0.97). Similarly, the Cronbach α values for the ASST scores were also of high values with regards to pain (0.89), disability (0.94), and total score (0.97) respectively. Comparing the scores between the first and the second use of the ASST revealed no statistically significant mean differences of -1.9 (95% CI—3.61 to 0.17).

**Conclusion:**

The Arabic-translated version of the SST showed high reliability, internal consistency, and construct validity based on substantial correlations of the ASST with Arabic SPADI and VAS. We recommend the Arabic version of the SST for the evaluation of Arabic-speaking patients with shoulder dysfunction.

## Introduction

The incidence of shoulder complaints in daily general practice is high. Shoulder pain is responsible for about 16 percent of all musculoskeletal complaints [1] and is considered the second most common musculoskeletal disorder in the primary care setting [2]. The yearly incidence of shoulder pain was reported as 15 new episodes per 1,000 patients seen in the primary care setting [3]. Although patients with shoulder disorders recover after some time, there is a probability of recurrence of pain [4]. Shoulder dysfunction may cause pain that hinders activities of daily living and leads to decreased shoulder mobility, thereby having a negative impact on the quality of life including both the physical and mental quality of life [5]. In addition to the negative influence on productivity and total number of working hours, it has a significant socioeconomic effect.

In general, valid assessment tools can assist at least in two important functions for the health care practitioner.

First, the results of such assessments will identify which patients and conditions benefit from the various interventions used. The second purpose of the outcome is to support clinical research to compare the treatment among different populations and to study the cost effectiveness of each treatment [6]. Therefore, it is desirable to have a valid, reasonable, and useful tool to be used by health care providers.

In recent years, the need for research to resolve shoulder dysfunction has been clearly identified based on patient needs and concerns, including clinical trials to evaluate therapeutic impact and cost effectiveness of treatments. The creation of validated patient-reported outcome measures (PROMs) has given clinical outcome assessment a new dimension [7]. PROMs are essential for determining the impact of patient complaints on their lives and allowing patients to self-evaluate the effects of interventions on their physical condition. However, the efficacy of these studies will be determined by the existence of valid shoulder outcome measures.

Using long questionnaires as one of the outcome measures, may decrease response rate and increase respondent fatigue and limit everyday use [8]. As a result, a number of useful shoulder scales have been created. The University of California-Los Angeles (UCLA) Shoulder Scale, the Simple Shoulder Test (SST), and the Shoulder Pain and Disability Index (SPADI) are three of the most widely used shoulder outcome measures.

Furthermore, the translation and validation of internationally used patient self-reported outcome scales would result in culturally similar instruments, allowing direct comparison of national and international study results [9].

The (SST) was originally published in 1993 in the conference proceedings of the American Association of Orthopedic Surgeons (AAOS) Symposium Series the shoulder; “A balance of mobility and stability”. The SST measures shoulder function from a patient’s perspective. It is composed of 12 questions that require a simple ‘yes’/’no’ response. The simplicity of the SST is often cited as one of its strength [10, 11].

In patients with shoulder dysfunction, the SST was described as a simple and fast patient-reported outcome that measured functional limitations of the affected shoulder. It takes 3 minutes to complete, and patients can do it at home, making it very convenient for busy clinician [12]. The severity of shoulder condition is examined by the compromise of activity of daily living functions. The answers to simple shoulder test questions deliver a consistent way of recording the function of a shoulder [13].

Till present, the SST has not yet been translated and validated in Arabic, which would seem to be a realistic alternative to having the therapist orally interpret the questions within the Arab population.

As most standard questionnaires and indexes were developed for English-speaking populations, however, since cultural groups differ in disease expression and their use of different healthcare services, there is a great need for interventions that are explicitly intended to be used in non-English speaking countries. With the increasing number of broad multicenter studies, the need will become much more apparent.

The shoulder disorder treatment needs arise from the compromised function and determining the success of the management intervention essentially depends on the patient’s ability to restore function.

The use of functional outcomes at random in physiotherapy departments in the United Arab Emirates certainly highlights the need for a shoulder functional scale to be validated and created for Arabic-speaking patients. For the sake of brevity and convenience, invalidated and untested Arabic adaptations of existing shoulder dysfunction scales are frequently used. However, the psychometric properties of such scales have yet to be determined. This requirement will most likely be met by a reliable, valid, and receptive adaptation of functional ability scale developed for patients with shoulder pain.

The objectives considered for this study were:

1. To cross-culturally adapt the Arabic version of the SST developed in the UAE to devise a pre-final SST Arabic version.
2. To test a pre-final SST Arabic version on a target group of patients with shoulder pain to create the final Arabic version.
3. To assess the final SST Arabic version’s reliability, internal consistency, and construct validity.

## Methods

### Study design

A cross-sectional observational design for cross-cultural adaptation and psychometric testing of the ASST.

### Setting

This study was conducted at Physiotherapy departments in hospitals under The Ministry of Health and Prevention, UAE.

### Participants and criteria

One hundred and forty patients with shoulder pain from different regions in the UAE were included in this study.

The ethical approval was obtained from the Ministry of Health and Prevention (MOHAP) (Approval Reference No: MOHAP/DXB-REC/JAA/No.98/2020). Prior to commencement of the study, a written informed consent was obtained from each participant.

A convenient sample of 140 patients having chronic shoulder pain (≥3 months) and dysfunction receiving treatment from the outpatient orthopedic departments in the Ministry of Health and Prevention Hospitals, UAE was considered. Patients were Emiratis aged 20 years or above, of both genders and were included if they had symptomatic shoulder pain and dysfunction and could read and understand Arabic.

Non-Emiratis, with a history of inflammatory joint diseases, rheumatoid arthritis, neck pathology, neurological diseases, diabetes mellitus, shoulder surgery, cardiac sources of shoulder pain and those unable to read and understand Arabic were all excluded. An estimated sample size of not less than 102 participants required for this study was calculated based on an expected Cronbach’s alpha value of 0.8 with significance level (α, two sided) = 0.05. An excess of 20% was considered for possible dropouts. However, the initial sample included 161 participants who volunteered to participate, but after initial screening, only 146 participants met the inclusion criteria. During the course of the study 6 participants dropped out due to personal reasons (3) or changing therapy provider (3). The remaining 140 participants were eligible and all joined our study.

### The SST

The SST is a series of 12 dichotomous (yes or no) questions about pain and function of the affected shoulder. The first two questions were about pain and the rest 10 question were about disability. The total SST score of pain is the number of yes responses and ranges from 0 (No pain) to 2 (extreme pain). The total SST score of disability is the number of yes responses and ranges from 0 (extreme limitations in physical function) to 10 (able to perform all functions). Results are reported as an overall score or as the percentage according to ability in performing each of the individual functions (S1 Appendix).

Before administering the Arabic SST, the participants were given a briefing on the process. During the initial interview, demographic information such as age, gender, height, weight, and body mass index (BMI) were registered, as well as from hospital medical records. The research was divided into two steps: first, SST was translated and adapted into Arabic following an international criteria for translation and adaptation [14]; second, the Arabic version was piloted, which was followed by an analysis of the questionnaire’s psychometric properties.

### Translation and cross-cultural adaptation

Two independent bilingual translators, fluent in both English and Arabic, whose first language was Arabic, translated the English version of SST into Arabic. One of the translators was a professional translator with no medical background, while the other was a professional translator specialized in the physiotherapy field. The first translator was aware of the method and objectives, while the second translator was blinded. The two translators compared their versions, addressed contradictions, and cleared any discrepancies in the translations after each produced a separate Arabic forward translation. Eventually, one version of the ASST was developed. The developed version of the ASST was then back translated into English by two translators. Similarly, one had a medical background and was aware of the purpose, whereas the other did not have any medical background and was blinded.

The ASST was piloted on ten patients with shoulder pain before being finalized. The pilot sample was eventually considered in the final analysis. The participants were comfortably seated while testing the Arabic version that had been cross-culturally adapted.

Participants were instructed to choose the appropriate point on the Arabic questionnaire’s sheet, which reflected their level of activity limitations and participation restrictions owing to shoulder pain. The patients were asked if they faced any difficulty understanding the translated SST.

A review committee comprising of two independent bilingual translators, fluent in both English and Arabic. One of the translators was a professional translator with no medical background, while the other was a professional translator specialized in a medical field (orthopedic surgeon). They were both blinded to the original English version and their task was to look over all the details from both the patients and the translators. The final version was then put through further testing to determine its validity and reliability in Emiratis with shoulder pain and dysfunction [14].

Translation and cross-cultural adaptation of SST required conversion of units of measure from pound to kilograms and yards to meters. One pound was converted in 0.5 kg while, Ten and 20 yards were converted and approximated to 9 and 18 meters respectively [15].

In addition, instead of using the softball which is not common in the UAE, instead we used tennis or baseball as terms of reference (S2 Appendix).

It was critical that the patients answer these questions independently to reflect their own assessment of how well his or her shoulder functions. Concerns about interobserver variability are eliminated because the patient is the consistent evaluator of their shoulder [16]. The SST measures the functional state of the shoulder rather than degrees of motion, radiograph appearance, or isokinetic torque measurements. We confirmed that almost all participants aged 60 to 70 years could perform the twelve basic functions before the Simple Shoulder Test was introduced into clinical practice.

### Psychometric testing

The ASST test–retest reliability was investigated by making the patient complete it during a second visit scheduled at least 48 hours after the initial session to reduce the likelihood of relevant changes in the patient’s clinical condition [17]. Both sessions were conducted by the same investigator and in the same treatment room. The construct validity of the ASST was tested by comparing its scores to the severity of shoulder pain measured using the visual analogue scale (VAS) and the Arabic version of SPADI [17].

### Statistical analyses

The data was analyzed using SPSS for Windows version 25 (IBM Inc., Armonk, NY, USA). Descriptive statistics were calculated for all variables. Scores of the SST as well as SPADI questionnaires were calculated on the basis of raw sum scores [17, 18].

Floor and ceiling effects were considered if more than 15% of participants achieved the lowest or highest scores respectively [19].

The test–retest reliability of the ASST, pain, disability, and total scores were assessed using intraclass correlation coefficients (ICC). Reliability was examined at 95% confidence interval (CI) levels. The absolute measurement error was estimated by calculating the SEM (SEM = s/√N), which was used to calculate the minimal detectable change (MDC) [MDC = 1.96 ×√2 × SEM] [20–22]. The Cronbach α value was calculated to examine internal consistency of the questionnaire [23].

The construct validity was assessed using Spearman rank coefficients between SST score, total pain, and disability score of the Arabic SPADI and the score of VAS.

The values were interpreted as: excellent relationship, >0.91; good, 0.90–0.71; moderate, 0.70–0.51; fair, 0.50–0.31; and little or none, < 0.30 [24]. The level of significance in all tests was set at p < 0.05.

## Results

One hundred and forty Emiratis (63 females and 77 males) participated in the study. The sample included people with a variety of diagnoses, with the most common being rotator cuff impingement. The characteristics of the participants are mentioned in Table 1. The mean age and standard deviation (SD) of the group were 39.3 and 4.9, respectively. Table 2 summarizes the baseline score of VAS, Arabic SPADI and Arabic SST.

**Table 1:** Participants’ characteristics.

Participants’ characteristics	Mean (SD)^a^
**Sex [no. (%)]**	
Male	60 (47.5)
Female	80 (52.5)
**Age (years)**	
Mean (SD)	39.3 (4.9)
Range	23–65
**Height (m)**	
Mean (SD)	1.62 (0.7)
Range	1.5–1.74
**Weight (kg)**	
Mean (SD)	75.5(8.6)
Range	55–102
**BMI (kg/m** ^**2**^ **)**	
Mean (SD)	26.4(4.1)
Range	18.5–40.6
**Diagnosis [no. (%)]**	
Frozen shoulder	35 (25)
Shoulder impingement syndrome	63 (45)
Acromioclavicular joint dysfunction	7 (5)
Shoulder Instability	28 (20)
Shoulder dysfunction with unknown cause	7 (5)

^a^SD: Standard deviation

**Table 2:** Descriptive baseline data.

Scale	Mean (SD)
^a^VAS	5.5 (3.1)
^b^ASST TOTAL SCORE	7 (1.2)
ASST PAIN SCORE	1 (1)
ASST DISABILITY SCORE	6 (2.1)
^c^SPADI TOTAL SCORE	47.2 (21.4)
SPADI PAIN SCORE	16.8 (10.2)
SPADI DISABILITY SCORE	29.2 (13.1)

^a^VAS: Visual Analogue Scale

^b^ASST: Arabic Simple Shoulder Test

^c^SPADI: Shoulder Pain and Disability index

No floor or ceiling effects were revealed from the responses of the participants.

In addition, the average time to complete the ASST was 102.4±32.5 seconds.

### Reliability and internal consistency

The Cronbach α values for the ASST score were high as reported; (0.89), (0.94) and (0.97) for pain, disability, and total score, respectively. Similarly, the ICCs for score of ASST were high; pain 0.84 (0.78–0.93), disability 0.96 (0.93–0.97) and total score 0.95 (0.91–0.97) as shown in Table 3. No statistically significant mean differences in scores were found between the first and the second use of the ASST -1.9 (95% CI—3.61 to—0.3). The SEM was reported as (7.4) based on repeated measurements for test–retest. The MDCs based on the SEM for test–retest was reported as (20.5).

**Table 3:** Test-retest reliability and agreement measures of the Arabic shoulder simple test data.

ICC (95%CI)		Mean difference	^standard deviation of the differences	SEM (95% CI)	MDC
**Pain score**	0.84(078–0.93)	- 1.06	5.22	3.27 (-7.61 to 4.97)	9.06
**Disability score**	0.96(0.93–0.97)	- 1.13	5.19	3.0 (-7.45 to 4.36)	8.31
**Total score**	0.95(0.91–0.97)	- 1.9	9.64	4.98 (-11.78 to 9.13)	13.8

CI: confidence interval;

^standard deviation of the differences;

MDC: minimal detectable change; SEM: standard error of measurement; based on the SD of the first trial (see Table 2)

### Validity

The construct validity was assessed using Spearman rank coefficients between SST score, total pain, and disability score of the Arabic SPADI and the score of VAS. There was a moderate positive correlation between the VAS and SST scores (r = 0.43) (P < 0.01). There was a strong positive correlation between the total Arabic SPADI score and SST score (r = 0.79) (P < 0.01), The SPADI pain score and SST score (r = 0.73) (P < 0.01), The SPADI disability score and SST score (r = 0.81) (P < 0.01) (Table 4).

**Table 4:** Correlation matrix of ASST pain, disability, and total scores versus VAS, SPADI pain, disability, and total scores.

	Pain score	Disability score	Total score
^a^VAS	0.43	0.79	0.61
^b^SPADI	0.73	0.81	0.77

All correlations were significant at P< 0.01 (Spearman rank coefficients).

^a^VAS: Visual Analogue Scale

^b^SPADI: Shoulder Pain and Disability index

## Discussion

Arabic translation and cross-cultural adaptation of the SST was chosen for several reasons: it is concise, self-administered, and measures symptoms and functional status with a focus on physical function at the level of shoulder dysfunction, physical impairments, activity limitations and participation restrictions in daily life. This study showed that the Arabic version of the SST is a valid, reliable, and internally consistent instrument for the assessment of patients with shoulder complaints.

The SST could be used to serve large populations to measure the impact of activity on life as reflected in shoulder function. It is available and validated in several languages and has been proven to be a reliable evaluation method in postsurgical conditions, musculoskeletal conditions, instability, and arthritis. We assumed that it was reasonable to correlate the ASST to the Arabic SPADI, which is already available and well correlated with the gold standard.

In many studies, SPADI has been correlated to other standard tools such as DASH and ASES. Since the SPADI has also been validated and correlated against the DASH [17, 25–29].

This research presents the process of cross-cultural adaptation and validation of the ASST and reported evidence of regarding its reliability and validity in patients with shoulder pain and dysfunction. Initially, the SST was translated and adapted into an Arabic version using internationally accepted guidelines [14]. Part of the translation process was a pilot study of the Arabic SST. Then, a detailed analysis of the psychometric properties of the simple shoulder test was performed.

The terms used in the Arabic translation were insistently simple and commonly used Arabic terms so that they could be used by another Arab population in any healthcare system. The test-retest reliability was good to excellent in both subscales as well as the overall total score. The reliability of the Arabic SST had an ICC value of 0.95 (0.91–0.97), which was higher than the English version of the SST (ICC = 0.65) but was almost similar to the Italian (ICC 0.97) (CIs 95%) [29] and Dutch versions (ICC = 0.92) (CIs 95%) [25]. Although the SEM of the Arabic SST came in agreement with Roddey et al. [30], the MDC was quite less (13.8) but we believe this might be a result of the smaller sample size in the current study (140) as compared with Roodey et al., which included 192 participants [30]. The internal consistency of the Arabic SST was higher (0.97) than that of the English version (0.85) and the Italian version (0.87). Similarly, in agreement with our findings, a previous study carried out by Van Kampen et al. showed that the internal consistency of the Dutch version of the SST (Cronbach’s α > 0.95) was high [31].

The construct validity was tested by comparing the ASST score with the VAS and the Arabic version of SPADI. As compared to data from the original and translated versions of the SST, the findings in this analysis were identical which came in agreement with Godfrey et al. [16]. Furthermore, Roach et al. [32] reported that all the SPADI items had at least moderate correlations with the first item of the SST. Previous studies have also compared SST scores with shoulder range of motion and found that patients with more pain and disability had less functionality [31, 32].

In addition, our study included VAS to assess shoulder pain intensity [33, 34]. The construct validity of the ASST revealed scores to be correlated to the intensity of shoulder pain and disability as measured by the VAS. This provides support for the ASST as well as new information on the relationships between pain intensity and SST pain reporting indicating that higher levels of reported disability were associated with greater pain intensity.

### Methodological considerations and future recommendations

Even though the study was comparable to the general population of patients with shoulder disorders in terms of the mean age and gender composition, it could differ on other important variables. For example, we did not collect information about the study population’s clinical/radiological diagnosis for shoulder pain or acuteness of condition, nor did we account for socioeconomic status or occupation or physical activity (athletes). Another point to note is that our research did not consider whether participants were medically insured or not. As a result, it is possible that our sample differed from the general population on these variables.

In addition, this study did not report the responsiveness nor the minimal clinically important differences. To complete the evaluation of the Arabic SST’s psychometric properties, a study investigating the sensitivity to change (responsiveness), and the minimal clinically important (perceptible) difference is recommended.

## Conclusion

The Arabic-translated version of the SST showed high reliability, internal consistency, and construct validity based on substantial correlations of the ASST with Arabic SPADI and VAS. We recommend the Arabic version of the SST for the evaluation of Arabic-speaking patients with shoulder dysfunction.

## Supporting information

S1 Appendix The Simple Shoulder Test Questionnaire (SST). (PDF)

S2 Appendix The Arabic Simple Shoulder Test Questionnaire (ASST). (PDF)
