# Supplementary material for: Cross-cultural adaptation and validation of the Arabic version of the simple shoulder test in the United Arab Emirates
Source: PLoS One. 2022 May 4;17(5):e0267885. doi: 10.1371/journal.pone.0267885 (PMC9067704; doi:10.1371/journal.pone.0267885)
Supplement: S1 Appendix — (PDF) [file pone.0267885.s001.pdf]

Simple Shoulder Test

Dominant Hand (fill in only one oval): Right ⬭ Left ⬭ Ambidextrous ⬭

Shoulder Evaluated (fill in only one oval): Right ⬭ Left ⬭

		Yes	No
1.	Is your shoulder comfortable with your arm at rest by your side?	⬭	⬭
2.	Does your shoulder allow you to sleep comfortably?	⬭	⬭
3.	Can you reach the small of your back to tuck in your shirt with your hand?	⬭	⬭
4.	Can you place your hand behind your head with the elbow straight out to the side?	⬭	⬭
5.	Can you place a coin on a shelf at the level of your shoulder without bending your elbow?	⬭	⬭
6.	Can you lift one pound (a full pint container) to the level of your shoulder without bending your elbow?	⬭	⬭
7.	Can you lift eight pounds (a full gallon container) to the level of your shoulder without bending your elbow?	⬭	⬭
8.	Can you carry twenty pounds at your side with the affected extremity?	⬭	⬭
9.	Do you think you can toss a softball under-hand twenty yards with the affected extremity?	⬭	⬭
10.	Do you think you can toss a softball over-hand twenty yards with the affected extremity?	⬭	⬭
11.	Can you wash the back of your opposite shoulder with the affected extremity?	⬭	⬭
12.	Would your shoulder allow you to work full-time at your regular job?	⬭	⬭

Office Use Only	DJD	SDJD	RA	FS	PTSS	AVN	CA	CTA	SA	PTCL	RCT	TUBS	AMBRII
	⬭	⬭	⬭	⬭	⬭	⬭	⬭	⬭	⬭	⬭	⬭	⬭	⬭

00000000 02 00 01 01 0
