# Supplementary material for: Cross-cultural adaptation and validation of the Arabic version of the simple shoulder test in the United Arab Emirates
Source: PLoS One. 2022 May 4;17(5):e0267885. doi: 10.1371/journal.pone.0267885 (PMC9067704; doi:10.1371/journal.pone.0267885)
Supplement: S2 Appendix — (PDF) [file pone.0267885.s002.pdf]

إختبار بسيط للكتف

اليد المهيمنة (إملأ إختيار واحد فقط): اليد اليمنى ( ) اليد اليسرى ( ) كلتا اليدان ( )

الكتف الذي تم فحصه: الأيمن ( ) الأيسر ( )

	نعم	لا
1- هل تشعر بالراحة إذا كانت ذراعك في وضع الراحة الي جانبيك؟	( )	( )
2- هل يسمح لك كتفك بالنوم بشكل مريح؟	( )	( )
3- هل يمكنك الوصول بيدك إلي أسفل ظهرك لإدخال قميصك بالبنطال؟	( )	( )
4- هل يمكنك وضع يدك وراء رأسك بحيث يكون كوعك بإتجاه مباشر إلي الجانبين؟	( )	( )
5- هل يمكنك وضع قطعة معدنية على رف يحازي مستوى كتفك دون الحاجة لثني كوعك؟	( )	( )
6- هل يمكنك رفع ما وزنه رطل واحد (0.5 كيلوجرام) أو ما يساوي حاوية نصف لتر إلي مستوى كتفك دون الحاجة لثني كوعك؟	( )	( )
7- هل تستطيع رفع ثمانية أرطال (3.6 كيلوجرام) أو ما يساوي حاوية جالون إلي مستوى كتفك دون الحاجة لثني كوعك؟	( )	( )
8- هل تستطيع حمل عشرين رطلاً (9 كيلوجرام) إلى جانبك بواسطة الطرف المصاب؟	( )	( )
9- هل تعتقد إنه يمكنك قذف الكرة اللينة (تشبه كرة البيسبول أو التنس) بحيث تكون اليد تحت مستوى الكتف لمسافة 18 متر أو60 قدم بواسطة الطرف المصاب؟	( )	( )
10- هل تعتقد إنه يمكنك قذف الكرة اللينة (تشبه كرة البيسبول أو التنس) بحيث تكون اليد فوق مستوى الكتف لمسافة 18 متر أو 60 قدم بواسطة الطرف المصاب؟	( )	( )
11- هل يمكنك غسل الجزء الخلفي من الكتف المعاكس بواسطة الكتف المصاب؟	( )	( )
12- هل تعتقد أن كتفك يمكنك من العمل بدوام كامل في عملك الدائم؟	( )	( )
